# Supplementary material for: Enhanced Immunomodulation, Anti-Apoptosis, and Improved Tear Dynamics of (PEG)-BHD1028, a Novel Adiponectin Receptor Agonist Peptide, for Treating Dry Eye Disease
Source: Pharmaceutics. 2022 Dec 26;15(1):78. doi: 10.3390/pharmaceutics15010078 (PMC9863990; doi:10.3390/pharmaceutics15010078)
Supplement: Supplementary file 1 [file pharmaceutics-15-00078-s001.zip › pharmaceutics-2013718-supplementary.pdf]

pharmaceutics

MDPI

Supplementary Materials: Enhanced Immunomodulation, Anti-apoptosis, and Improved Tear Dynamics of (PEG)-BHD1028, a Novel Adiponectin Receptor Agonist Peptide, for Treating Dry Eye Disease

In-Kyung Lee [1], Kyung-Chul Yoon [2], Seong-Soo Kang [3], Su-Kyung Seon [3], Kwanghyun Lee [4] and Brian B Kim [1,*]

Table S1. Mean value of diagnostic tests for each item group in mice (Mean ± SD).

Mice model	After inducing dry eye	EDE	Vehicle (PBS)	(PEG)-BHD1028		
				0.001%	0.01%	0.1%
Tear volume (μL)	Day 5	0.019 ± 0.002	0.020 ± 0.004	0.024 ± 0.003	0.026 ± 0.003	0.028 ± 0.002
	Day 10	0.016 ± 0.002	0.018 ± 0.002	0.021 ± 0.002	0.027 ± 0.003	0.032 ± 0.004
TBUT (sec)	Day 5	1.304 ± 0.086	1.324 ± 0.114	1.333 ± 0.081	1.406 ± 0.082	1.408 ± 0.095
	Day 10	1.193 ± 0.092	1.229 ± 0.060	1.359 ± 0.140	1.530 ± 0.137	1.483 ± 0.113
CFS (score)	Day 5	10.875 ± 2.357	10.250 ± 2.435	9.875 ± 1.808	8.625 ± 1.598	8.125 ± 1.553
	Day 10	12.375 ± 1.923	12.625 ± 1.302	11.000 ± 1.927	7.625 ± 1.598	8.375 ± 1.302

Table S2. Mean value of diagnostic tests for each item group in rabbits (Mean ± SE).

Rabbit model	After inducing dry eye	Vehicle	(PEG)-BHD1028			Cyclosporine 0.05%
			0.1%	0.2%	0.4%	
STT-2 (mm)	Baseline	9.83 ± 1.74	6.6 7± 0.96	8.50 ± 1.39	9.17±1.23	8.33 ± 0.38
	Day 0(EDE)	2.67 ± 1.10	3.00 ± 1.22	3.33 ± 1.28	3.33±1.22	2.50 ± 1.10
	Day 5	1.17 ± 0.50	4.67 ± 0.65	3.33 ± 0.51	4.33±0.45	4.17 ± 0.37
	Day 10	2.17 ± 0.64	6.50 ± 0.87	7.50 ± 0.99	6.67±1.02	4.33 ± 0.45
TBUT (sec)	Baseline	48.33 ± 3.88	49.67 ± 3.17	49.83 ± 2.10	53.00 ± 2.29	52.00 ± 2.25
	Day 0(EDE)	8.20 ± 0.51	8.50 ± 1.0	8.89 ± 1.34	8.22 ± 1.16	8.92 ± 1.34
	Day 5	8.81 ± 1.20	10.64 ± 0.99	15.68 ± 1.58	19.88 ± 1.42	11.19 ± 1.17
	Day 10	10.22 ± 1.97	17.88 ± 1.91	17.72 ± 1.74	22.71 ± 1.87	11.49 ± 2.23
CSS (score)	Baseline	0.33 ± 0.19	0.00 ± 0.00	0.00 ± 0.00	0.00 ± 0.00	0.17 ± 0.15
	Day 0(EDE)	8.75 ± 0.46	6.67 ± 0.30	9.17 ± 0.37	8.00 ± 0.62	6.05 ± 1.20
	Day 5	8.50 ± 0.89	7.17 ± 1.21	6.83 ± 0.64	5.67 ± 0.38	5.67 ± 0.81
	Day 10	12.50 ± 1.24	6.67 ± 0.56	6.17 ± 0.55	3.83 ± 0.64	6.17 ± 1.04

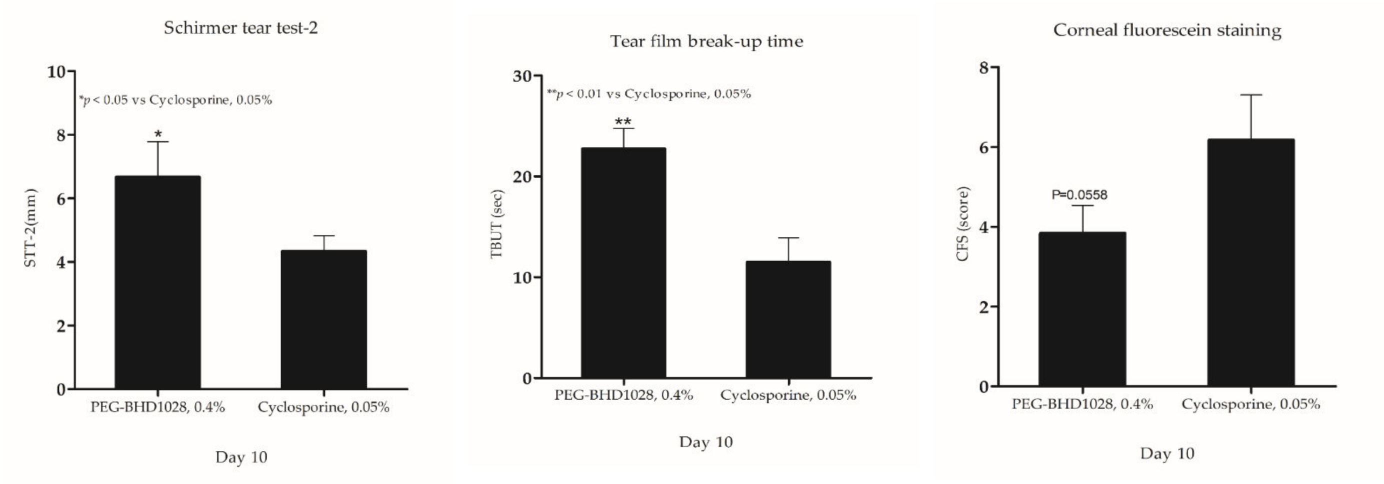

Figure S1. The results were expressed as means ± S.E.M. and were considered significant when $p < 0.05$. Statistical analysis was performed using Student's t-test by the computer program Prism (GraphPad Software, USA). Results are presented as means ± SEM ($n = 6$). ** $p < 0.01$ and * $p < 0.05$ vs positive control.

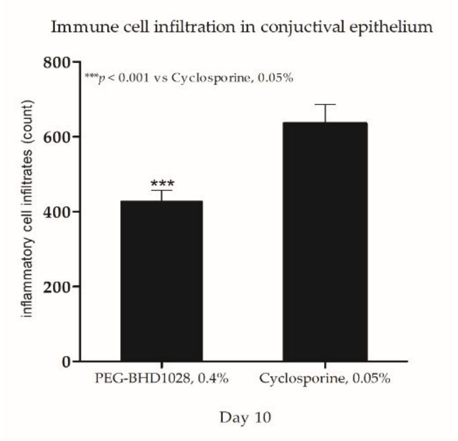

Figure S2. The results were expressed as means ± S.E.M. and were considered significant when $p < 0.05$. Statistical analysis was performed using Student's t-test by the computer program Prism (GraphPad Software, USA). Results are presented as means ± SEM ($n = 6$). *** $p < 0.001$ vs positive control.

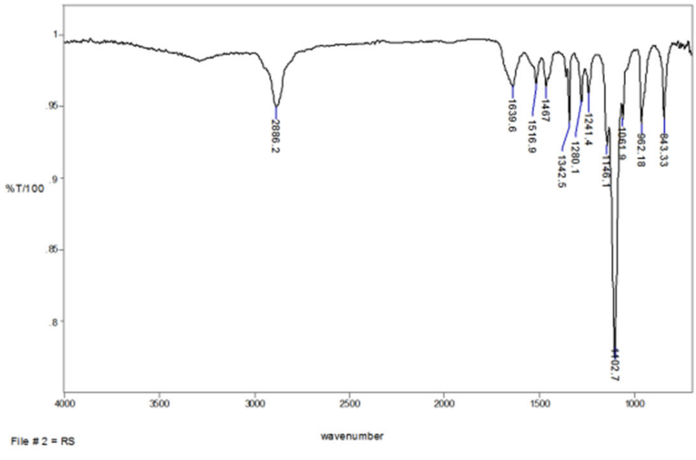

Figure S3. The conformational analysis of (PEG)-BHD1028 using Fourier transform IR (FT-IR) spectroscopy.

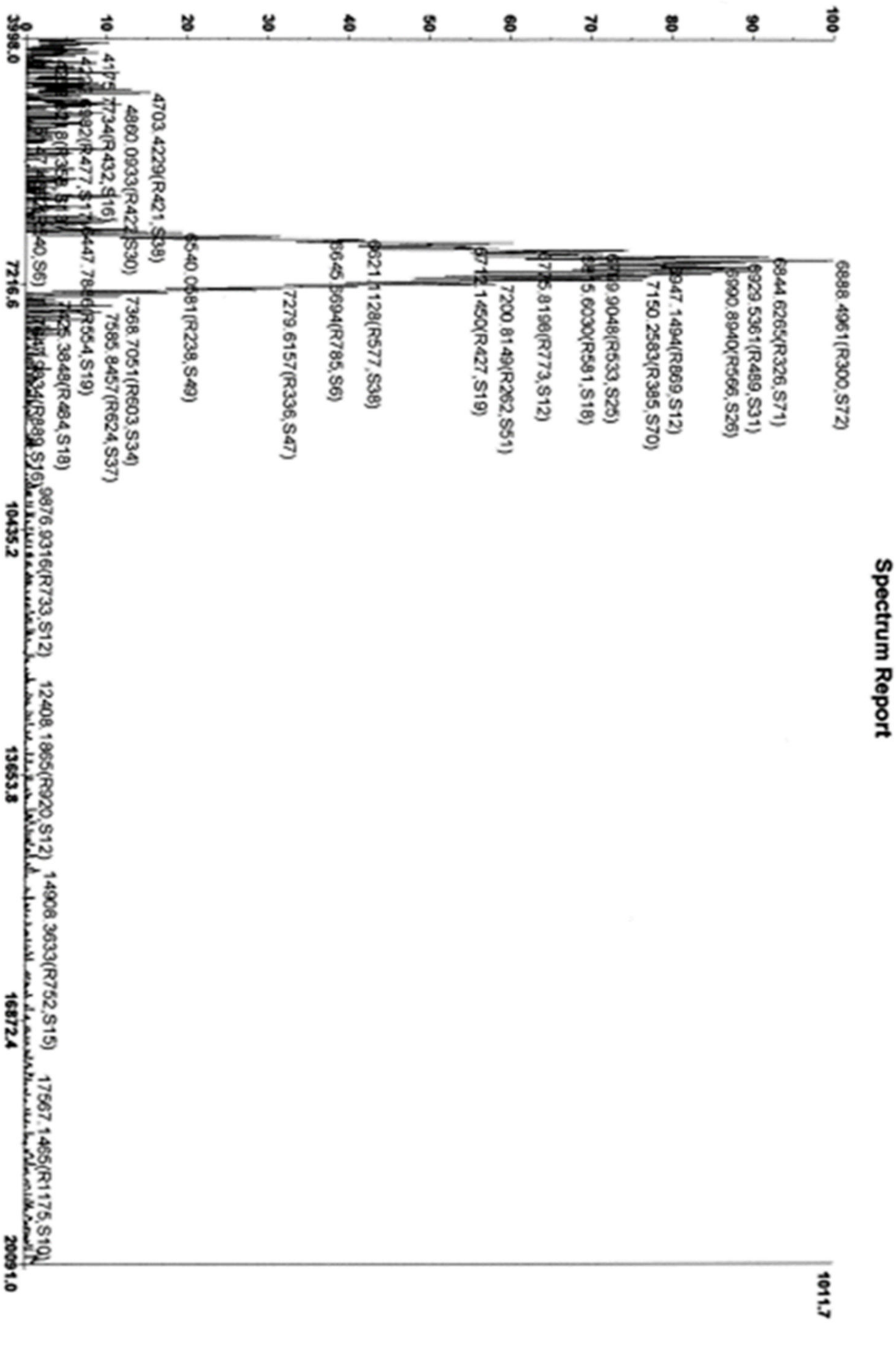

Figure S4. The identification of (PEG)-BHD1028 by MALDI-TOF mass spectrometry.
